# The challenge to improve the response of biomaterials to the physiological environment

**DOI:** 10.1093/rb/rbw012

**Published:** 2016-03-10

**Authors:** Nicholas A. Peppas, John R. Clegg

**Affiliations:** ^1^McKetta Department of Chemical Engineering,; ^2^Department of Biomedical Engineering,; ^3^Institute for Biomaterials, Drug Delivery and Regenerative Medicine; ^4^Department of Surgery and Perioperative Care, Dell Medical School and; ^5^Division of Pharmaceutics, College of Pharmacy, University of Texas at Austin.

**Keywords:** drug delivery, nanobiomaterials, regenerative mechanism, materials structure

## Abstract

New applications of biomaterials often require advanced structures containing synthetic and natural components that are tuned to provide properties unique to a specific application. We discuss how structural characteristics of biomaterials, especially hydrophilic ones, can be used in conjunction with non-ideal thermodynamics to develop advanced medical systems. We show a number of examples of biocompatible, intelligent biomaterials that can be used for organ replacement, biosensors, precise drug delivery over days or weeks, and regenerative medicine.

A number of challenges have been identified that are associated with the new generation of biomaterials. These challenges emanate from a series of changes or requirements in the field. The China–US Forum on Grand Challenges for Biomaterials in the 21st century held in Chengdu, China in November 2014 was a vehicle of identification of important subjects in the field. Indeed, in more general terms, the changing world of biomaterials, biomolecular engineering, therapeutics and drug delivery requires:

- Formation and fabrication of supramolecular assemblies comprising natural biological elements, structures or membranes;
- Synthesis and preparation of modified biological molecules;
- Biomolecular design of nanostructures, molecular adhesives; and
- Micropatterned and microfabricated arrays.

But at the same time, the changing world of therapy requires that a number of prerequisites be fulfilled for new biomaterials and products to enter the market:

- Lower cost;
- Simpler use of medical products;
- Product safety; and
- Early diagnosis.

We believe that a major new challenge in the field is the development of intelligent biomaterials that will be responsive to the surrounding biological fluid. In act, here we address the development and use of intelligent biopolymers such as hydrogels. Their idea is based on simple physicochemical characteristics first observed almost 70 years ago. For example, a pH-sensitive biomaterial is the simplest possible form of an intelligent system. Here, each fixed ion has an associated mobile counterion, which is restricted to remain in the gel. Osmotic pressure gives rise to swelling when these ions are dissociated. Similar behavior is observed with temperature-sensitive hydrogels.

These ideas are associated with new material needs imposed by the advance of Precision Medicine. This term denotes an emerging approach for disease prevention and treatment that takes into account an individual’s variations in genes, environment and lifestyle (an NIH definition slightly adopted here). Customization of healthcare with medical decisions, practices, and products may be tailored to the individual patient. Such approaches include:

- Targeted therapy;
- Precision surgery;
- Molecular imaging;
- Systems biology and medicine; and
- Nanomedicine.

We are therefore concluding that the changing world of disease treatment and health care leads to implications in biomaterials, regenerative medicine, drug delivery and intelligent medical devices Some of the advanced biopolymer-based systems of these developments are:

- Formation and fabrication of supramolecular assemblies;
- Modified biological molecules;
- New systems for targeting using nanoparticles;
- Protein delivery with patient preferred formulations;
- Intracellular delivery;
- Bionanotechnology;
- Systems for diagnosis, recognition and treatment;
- Intelligent biomaterials;
- Advanced methods of growth of new organs; and
- Advanced regenerative medicine.

## Biomolecule responsive hydrogels

**Figure 1.**
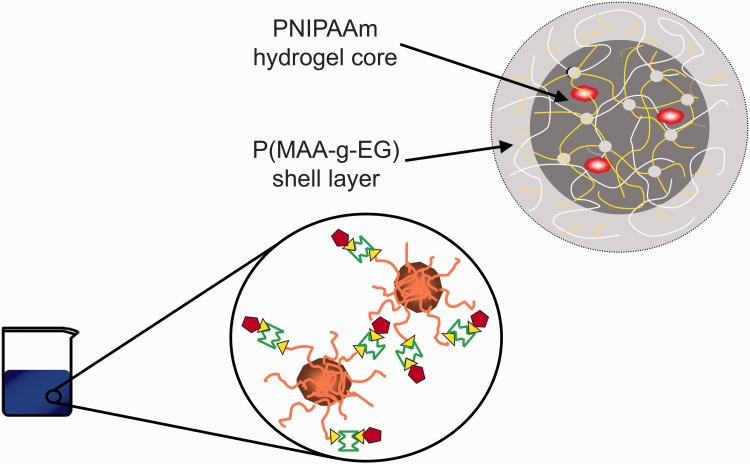
A hydrogel in nanoparticulate form based on P(MAA-g-EG) crosslinked and responding to the surrounding environment through capture of peptides, proteins, lipids, ions etc, via a set of sensitive tethers containing carbohydrates or other modified structures that will provide external recognition.

There is an immediate need to construct next-generation materials that recognize and respond to molecular cues for medical applications in biosensing, drug delivery and regenerative medicine. Fluctuation of a biomarker concentration *in vivo* is often indicative of a need for therapeutic intervention in diseased patients. Rather than having a physical change in polymer architecture, as a result of a more general environmental change such as temperature or pH, these smart materials identify and respond to the presence of single biomarkers in complex solutions. To enable this activity, it is necessary to synthesize rational network polymers that take motivation from the shape complementarity, electrostatic interactions, and hydrogen bonding exhibited by highly specific protein interactions *in vivo*. Figure 1 shows one such hydrogel in nanoparticulate form based on poly(methacrylic acid-g-ethylene glycol) (P(MAA-g-EG)) crosslinked and responding to the surrounding environment through capture of peptides, proteins, lipids and ions, via a set of sensitive, custom made tethers containing carbohydrates or other modified structures that will provide external recognition.

## Recognitive molecularly imprinted polymers

Molecularly imprinted polymers (MIPs) are a promising recognitive biomaterial, capable of recognizing, capturing and responding to biomarkers. MIPs were first generated as improved sorbents for affinity chromatography and proved capable separating template molecules from complex solutions [1]. Because of MIPs’ impressive ability to segregate chiral synthetic molecules, ease-of-production, environmental stability and cost effectiveness, the extension of MIPs into medical applications is quite promising.

**Figure 2.**
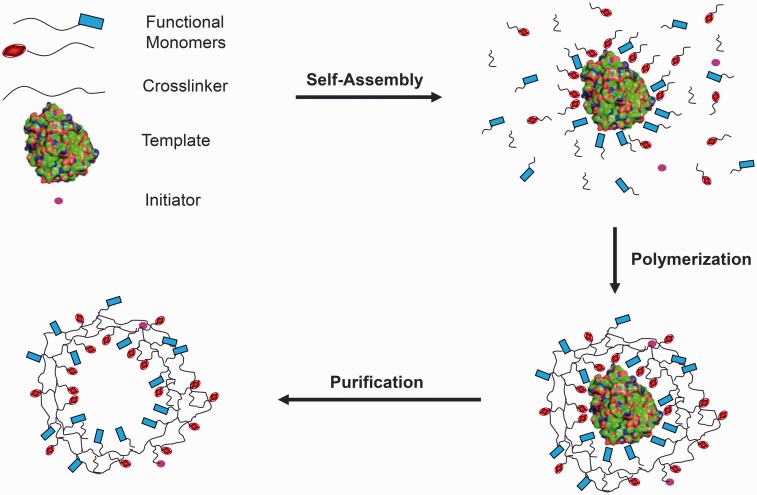
Step involved in the imprinting process of a template into a polymerizable material that leads to a three dimensional network with nanocavities that may again recognize the original template.

**Figure 3.**
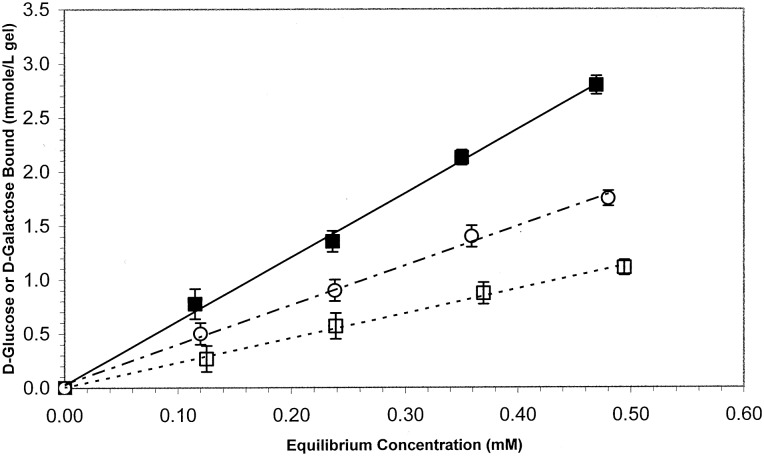
Recognitive behavior to D-glucose of a network imprinted with D-glucose (filled square) over D-galactose (open circle) and the control, non-imprinted material (open square).

Non-covalent MIPs are formed through the synthesis of functional monomers possessing anionic, cationic, hydrophilic or hydrophobic character into a crosslinked network around a template molecule. In a critical pre-polymerization step, functional monomers are allowed to complementarily self-assemble around exposed template moieties. Following polymerization and extraction of the template (see Fig. 2), a recognitive cavity possessing the geometric and chemical footprint of the template remains. These engineered cavities exhibit enhanced binding characteristics for template, in comparison to alternative diverse biomolecules. MIP performance is typically assessed by quantifying the mass binding capacity of MIPs for template, in comparison to non-imprinted polymers (NIPs), which are synthesized in the same manner as MIPs, excluding template. For example, Figure 3 shows the recognitive behavior to D-glucose of a network imprinted with D-glucose (filled square) over D-galactose (open circle) and the control, non-imprinted material (open square).

## MIP detection of small molecules

The capture and detection of small biological analytes using MIPs was a logical extension of preliminary chromatography applications. As these small molecules, such as glucose [2–4], cholesterol [5] or antimicrobials [6] pose few diffusional limitations within highly crosslinked networks[7]. Initial studies by Byrne *et al.* [4] identified that recognitive polyacrylamide networks crosslinked with poly(ethylene glycol) dimethacrylate were capable of recognizing D-glucose. As the mole percent of crosslinking monomer was increased, or length of the PEG linker was decreased MIPs bound significantly more D-glucose than NIPs. These networks were also capable of discriminating between D-glucose and D-galactose, exhibiting a 160% greater equilibrium association constant for glucose than the structurally similar D-galactose.

Further studies have investigated the imprinting of alternative small biomolecules in functional hydrogels. Spizzirri and Peppas synthesized cholesterol-imprinted networks comprised of methacrylic acid and poly(ethylene glycol) and specifically investigated the impact of solvent selection and network mesh of recognitive properties [5]. Polar aprotic solvents such as dimethyl sulfoxide (DMSO) or tetrahydrofuran (THF), which do not compete in hydrogen bonding, promoted such polymer-cholesterol interactions. Additionally, augmenting the network mesh size with porogens reduced the lag time for cholesterol recognition. Continued studies demonstrated that recognitive networks possessing functional complementarity to template epitopes are capable of molecular recognition [4]. This finding, in conjunction with knowledge that highly porous structures can maintain recognitive properties motivated the concept of imprinting larger, complex biomolecules.

## Protein MIPs

Proteins templates present many design limitations that contrast with small molecule imprinted networks. Protein diffusional considerations limit bulk MIPs to much lower crosslinking densities than small-molecule MIPs [8]. Additionally, polymerizations must be carried out under dilute monomer conditions in aqueous buffer as to retain native protein structure [9]. Each of these requirements conflicts with the conditions determined to increase polymer-template specificity discovered in the development of small-molecule MIPs.

**Figure 4.**
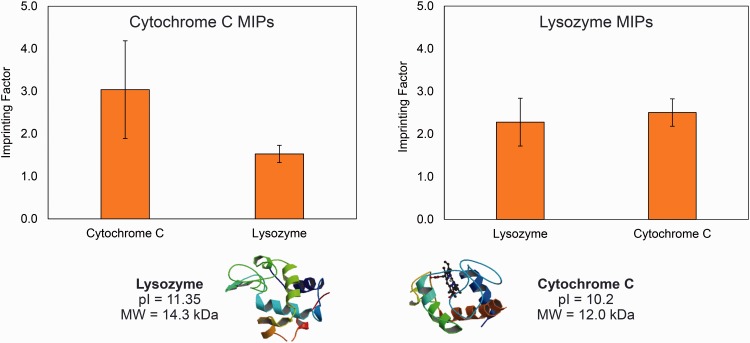
Recognition of cytochrome c cover lysozyme from a cytochrome c imprinted network.

Despite these challenges, protein MIPs are an active area of biomedical research because of the ultimate novelty of these ‘plastic antibodies’ [10]. Recognitive networks for cytochrome c, lysozyme [11], angiotensin II [12] and albumin [8] have been synthesized in synthetic and natural [13] hydrogel platforms. Recognition of cytochrome c is shown in Figure 4 from the recent studies of these authors. Several trends have emerged in these studies. Protein MIP networks are typically comprised of multiple functional monomers in order to match with diverse functionality present in protein epitopes [14]. Overcoming the diffusional limitations of large proteins led to the development of innovative surface-imprinting techniques [15]. Additionally, structural similarity within families of proteins has instigated selectivity of some MIPs for groups of similar, rather than single proteins [13]. Although this reality has complicated the development of MIPs with absolute selectivity for single proteins, it has also enabled the development of imprinted materials with affinity for diverse proteins using rationally selected of engineered templates.

## Applications in biosensing, drug delivery and regenerative medicine

**Figure 5.**
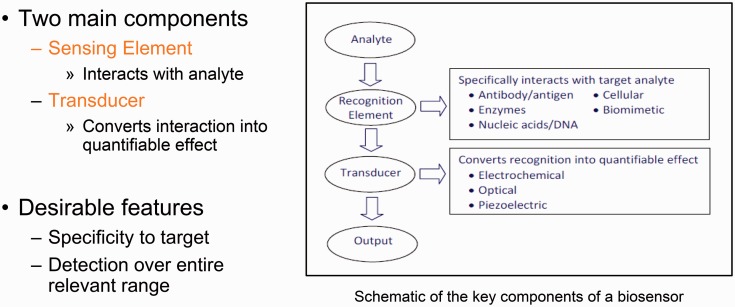
Schematic of the key components of a biosensor.

**Figure 6.**
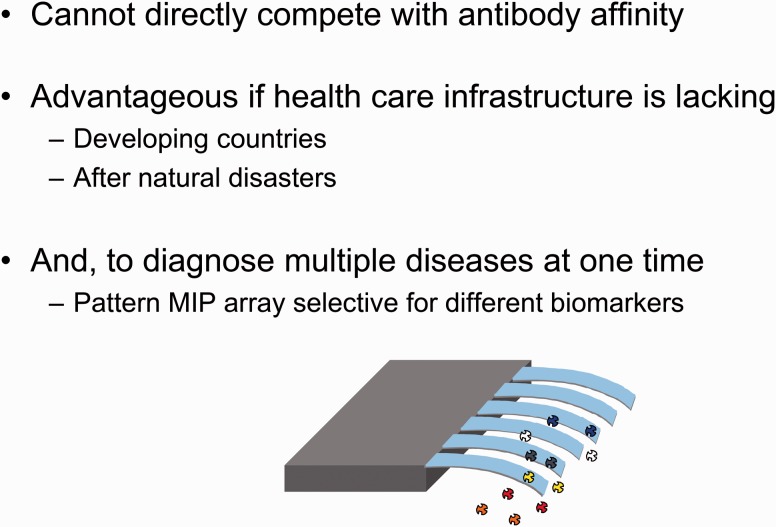
Recognition of multiple analytes identifying specific diseases by a single MIP-based biosensor.

MIPs have been employed to recognize desirable or undesirable biological compounds and consequently transduce a detectable signal. This is clearly shown in Figure 5 that shows the main characteristics of a biosensor. For example, glucose MIP networks have been synthesized on the surface of silicon microcantilevers, which promptly recognize glucose and transduce a signal through beam deflection [2, 16]. Such synthetic systems have been proposed as cost-effective, robust diagnostic platforms (Fig. 6). In addition to addressing needs in point-of-care diagnostics with low-cost, disposable MIP sensors, MIPs have been employed in a variety of sophisticated diagnostic platforms. MIPs have been synthesized on the surface of quartz crystal microbalance [17] and surface plasmon resonance [18] sensors in order to extract and detect nanomolar concentrations of analytes from biological samples. MIPs have also been employed as signal transducers in sensor arrays to quantify multiple analytes in a single assay [19].

Small molecule and protein MIPs have been employed for a number of purposes in drug delivery. High affinity MIP-template interactions can promote retention of loaded small-molecule drugs, sustaining delivery. These enhanced retention and diffusion properties have been employed in studies investigating therapeutic hydrogel contact lenses [20, 21]. As an alternative responsive delivery mechanism, MIP hydrogels are imprinted with a disease biomarker but loaded with a structurally similar therapeutic. As these drug-loaded gels equilibrate in the presence of the imprinted biomarker, biomarkers will compete for MIP recognitive pores, displacing the entrapped therapeutic. Such a system was employed to release hydrocortisone from 2-hydroxyethylmethacrylate hydrogels in response to testosterone binding [22, 23].

As an alternative to utilizing MIP recognition as a mechanism for retaining or releasing a therapeutic, thin MIP layers can serve as targeting elements on the surface of drug delivery vehicles. To this aim, Zhang *et al.* [24] synthesized p32 MIPs through an epitope imprinting approach. In addition to being able to selectively detect p32 at nanomolar concentrations, nearly three times as many MIPs were uptaken by p32 expressing cells as compared with NIPs. Such cell-specific MIPs, synthesized on the surface of pre-existing drug delivery vehicles, could behave similarly to alternative targeted nanomedicines.

In the future, MIP systems can be applied in responsive scaffolds for tissue engineering. Building off of the discoveries in protein and small-molecule MIPs, these smart materials can target lineage-dependent extracellular receptors to promote spatial docking and retention of desired cell types. MIPs could act as reservoir for the sustained release of growth factors within the bulk of a scaffold, or conversely as a depot to capture and retain signaling molecules secreted by active cells.

## A bright future for smart polymers

As knowledge in biology and medicine continues to evolve, scientists will become aware of ever-increasing physiological cues in diseased states. These cues may be generalizable to a condition, or identified on a single-patient basis. As this information becomes available however, the Grand Challenge will be to harness these molecular cues as triggers for therapeutic intervention. These materials may need to be responsive to general environmental changes in temperature or pH. Instead, responsiveness to single-molecules such as a sugar, small-molecule drug, proteins or abnormal cell may be appropriate. In all likelihood the answer will lie at the synergy of these fields. The diversity of smart polymer systems will ultimately need to match the great variety in medical conditions facing a multitude of patients.

The advantage presented by many of the pre-existing pH, temperature and molecularly responsive hydrogel materials lies in their ease of production and cost-effectiveness as ultimately, a major challenge for any diagnostic or therapeutic system lies in patient accessibility. It is paramount that the programmed response of an engineered biomaterial to its environment, which consequently delivers a therapeutic or aids in the diagnosis of disease, proceed reliably and reproducibly under practical shipment, storage and usage conditions. Fabrication of such robust and inexpensive biomaterial systems will enable treatments to be accessible to the masses worldwide.

## References

[1] Polyakov MV. Adsorption properties and structure of silica gel. Zhur Fiz Khim 1931;2:799–805.

[2] Hilt JZ Byrne ME Peppas NA. Microfabrication of intelligent biomimetic networks for recognition of D-glucose. Chem Mater 2006;18:5869–75.

[3] Oral E Peppas NA. Hydrophilic molecularly imprinted poly (hydroxyethyl-methacrylate) polymers. J Biomed Mater Res A 2006;78:205–10. 16602126 10.1002/jbm.a.30725

[4] Byrne ME Hilt JZ Peppas NA. Recognitive biomimetic networks with moiety imprinting for intelligent drug delivery. J Biomed Mater Res A 2008;84:137–47. 17600334 10.1002/jbm.a.31443

[5] Spizzirri UG Peppas NA. Structural analysis and diffusional behavior of molecularly imprinted polymer networks for cholesterol recognition. Chem Mater 2005;17:6719–27.

[6] Rodríguez E Navarro-Villoslada F Benito-Peña E Multiresidue determination of ultratrace levels of fluoroquinolone antimicrobials in drinking and aquaculture water samples by automated online molecularly imprinted solid phase extraction and liquid chromatography. Anal Chem 2011;83:2046–55. 21338057 10.1021/ac102839n

[7] Byrne ME Oral E Hilt JZ. Networks for recognition of biomolecules: molecular imprinting and micropatterning poly (ethylene glycol)-containing films. Polym Adv Technol 2002;13:798–816.

[8] Kryscio DR Peppas NA. Surface imprinted thin polymer film systems with selective recognition for bovine serum albumin. Anal Chim Acta 2012;718:109–15. 22305905 10.1016/j.aca.2012.01.006

[9] Kryscio DR Fleming MQ Peppas NA. Protein conformational studies for macromolecularly imprinted polymers. Macromol Biosci 2012;12:1137–44. 22777744 10.1002/mabi.201200068

[10] Hoshino Y Kodama T Okahata Y. Peptide imprinted polymer nanoparticles: a plastic antibody. J Am Chem Soc 2008;130:15242–3. 18942788 10.1021/ja8062875

[11] Bergmann NM Peppas NA. Molecularly imprinted polymers with specific recognition for macromolecules and proteins. Prog Polym Sci 2008;33:271–88.

[12] Lauten EH Peppas NA. Intelligent drug release using molecular imprinting methods: recognitive systems for angiotensin II. J Drug Deliv Sci Technol 2009;19:391–9.

[13] Bayer CL Herrero ÉP Peppas NA. Alginate films as macromolecular imprinted matrices. J Biomater Sci Polym Ed 2011;22:1523–34. 20633323 10.1163/092050610X514115

[14] Kryscio DR Peppas NA. Critical review and perspective of macromolecularly imprinted polymers. Acta Biomater 2012;8:461–73. 22100344 10.1016/j.actbio.2011.11.005

[15] Shi H Tsai WB Garrison MD Template-imprinted nanostructured surfaces for protein recognition. Nature 1999;398:593–7. 10217142 10.1038/19267

[16] Hilt JZ Gupta AK Bashir R Ultrasensitive biomems sensors based on microcantilevers patterned with environmentally responsive hydrogels. Biomed Microdevices 2003;5:177–84.

[17] Reimhult K Yoshimatsu K Risveden K Characterization of QCM sensor surfaces coated with molecularly imprinted nanoparticles. Biosens Bioelectron 2008;23:1908–14. 18374557 10.1016/j.bios.2008.02.011

[18] Wegner GJ Lee HJ Corn RM. Characterization and optimization of peptide arrays for the study of epitope-antibody interactions using surface plasmon resonance imaging. Anal Chem 2002;74:5161–8. 12403566 10.1021/ac025922u

[19] Greene NT Shimizu KD. Colorimetric molecularly imprinted polymer sensor array using dye displacement. J Am Chem Soc 2005;127:5695–700. 15826210 10.1021/ja0468022

[20] Ali M Horikawa S Venkatesh S Zero-order therapeutic release from imprinted hydrogel contact lenses within in vitro physiological ocular tear flow. J Control Release 2007;124:154–62. 17964678 10.1016/j.jconrel.2007.09.006

[21] Hiratani H Alvarez-Lorenzo C. Timolol uptake and release by imprinted soft contact lenses made of N, N-diethylacrylamide and methacrylic acid. J Control Release 2002;83:223–30. 12363448 10.1016/s0168-3659(02)00213-4

[22] Sreenivasan K. On the application of molecularly imprinted poly (HEMA) as a template responsive release system. J Appl Polym Sci 1999;71:1819–21.

[23] Sreenivasan K Sivakumar R. Imparting recognition sites in poly (HEMA) for two compounds through molecular imprinting. J Appl Polym Sci 1999;71:1823–6.

[24] Zhang Y Deng C Liu S Active targeting of tumors through conformational epitope imprinting. Angew Chem Int Ed 2015;54:5157–60. 10.1002/anie.201412114 25727886

